# Physical and chemical properties of the soils in selected communal property associations of South Africa

**DOI:** 10.7717/peerj.13960

**Published:** 2022-10-19

**Authors:** Malizo Ntalo, Khuliso Emmanuel Ravhuhali, Bethwell Moyo, Ntuwiseni Emile Mmbi, Kwena Hilda Mokoboki

**Affiliations:** 1 Food Security and Safety Niche Area, North-West University, Mahikeng, North West Province, South Africa; 2 Department of Animal Science, School of Agricultural Sciences, North-West University, Mahikeng, North West Province, South Africa; 3 Department of Animal Production, Fort Cox Agriculture and Forestry Training Institute, Middledrift, Eastern Cape, South Africa; 4 Department of Agriculture and Rural Development, Veld and Pasture Component, Tawoomba Agricultural Research Station, Bela-Bela, Limpopo Province, South Africa

**Keywords:** Soil type, Soil minerals, Livestock, Grazing, Vegetation cover

## Abstract

Communal Property Associations (CPAs) rangeland users need more knowledge on the state of their respective grazing lands and also the interaction of soil properties with grazing management implemented. This study aimed to investigate the effect grazing has on the physical and chemical properties of four different soil types found in selected CPAs of the Bela-Bela municipality, they are as follows: Mawela (Hutton-clay loam: HCL), Bela-Bela (Hutton-clay: HC), Moretele (Hutton-loamy sand: HLS) and Ramorula (Ecca sand-clay loam: ESCL).The macro and micro minerals, pH, nitrate-nitrogen, ammonium-nitrogen, organic carbon, soil particle size distribution, acidity and resistance were all measured. All data were subjected to two-way factorial analysis of variance (SAS, 2010). The topsoil was sampled at a depth of 300 mm at an interval of 100 m (100 and 200 m) from the same transect used for woody species data collection resulting in a total of 18 samples per CPA. In each CPA, three camps were selected. In each camp, three transects 200 m apart at the length of 200 m were set. In each transect, soils were drawn at 0, 100 and 200 m making a total of nine soil samples per each camp. The highest (*P* < 0.05) pH (7.14) recorded on the sub-soil was in HLS. Nitrate nitrogen (2.4 mg/kg) concentration on the topsoil was high (*P* < 0.05) in HC soil type. Soil organic carbon for both topsoil (0.66%) and subsoil (0.41%) was significantly lower (*P* < 0.05) in HLS soil type and ESCL soil type respectively. Phosphorus concentration was significantly high (*P* < 0.05) in ESCL soil type for both topsoil (12.86 mg/kg) and sub-soil (1.59 mg/kg). Iron concentration was high in both topsoil (11.8 mg/kg) and sub-soil (7.3 mg/kg) in ESCL soil type. Sub-soil manganese concentration was found to be higher (*P* < 0.05) in ESCL soil type (7.58 mg/kg). Soil resistance (2880 Ω ) measured in topsoil was high (*P* < 0.05) in HCL soil type compared to other soil types. Moreover, for the sub-soil the highest (*P* < 0.05) resistance (least salts) (3640 Ω) was recorded in ESCL soil type. For most of the soil types, the mineral concentration was higher in topsoil than in sub-soil, this trend explains that the uptake of these minerals by plants took place due to the inconsistencies of grazing management employed in these selected CPA farms. It is of colossal significance to properly manage rangelands, to allow a fair-to-good herbaceous layer in the presence of minerals in the soils and farmer should prioritize having enclosures and keeping N-fixing tree species in the rangelands to achieve the above mentioned conditions.

## Introduction

The essential function of Communal Property Associations (CPAs) in resource governance is increasingly being recognized in previously conducted studies (Ghate & Nagendra, 2005; Sebola & Mamabolo, 2020). Moreover, in communal property associations individuals have authority over resources and therefore common property organizations vary considerably from open access, which lack resource use rights (Agrawal, 2001). The management of grazing in CPAs is crucial for the maintenance of rangeland production and health. Studies have shown that there is a dynamic relationship that exists between the rangeland vegetation and soil properties (Herrick & Wander, 2018; Egeru et al., 2019). The features of any soil type play a role in the widely recognized resilience in semi-arid grazing lands, because they provide a degree of flexibility to the soil, in the notion that they provide pliancy to the soil, thus preventing disturbances in the biological system (Dougill, Heathwaite & Thomas, 1998; Walker & Meyers, 2004; Vetter, 2013). Rangeland health is predominantly dependent on the interaction between the soil and plant communities (Balestrini et al., 2015). Continuous grazing by livestock on the rangeland leads to poor physical, chemical and biological properties of soil, resulting in a dramatic change in vegetation and nutrient cycling (Lavado, Sierra & Hashimoto, 1996; Bolo et al., 2019). When such a problem is encountered in rangeland, the growth of perennial decreaser grasses will decline, and change in the herbaceous layer species and woody species establishment will occur (Ash et al., 2011). Undesirable land changes in livestock production as noted in studies conducted by Ash, Smith & Abel (2002), Holecheck et al. (2003) and Munyai (2012) give rise to constrains in management, this is due to the unavailability of instant drive grazing strategies.

The variation in soil properties influence the growth and development of both grasses and trees, soil depth impacts how deep or shallow roots of these plants can grow (Stichler, 2002). Furthermore, vegetation change and soils’ reaction to grazing pressure can be used as the most reliable indicator of rangeland degradation (Wang & Wesche, 2016a; Wang & Wesche, 2016b; Cao et al., 2018). Several studies highlighted the impact that grazing had on soil fertility (Wang & Batkhishig, 2014; Rahmanian et al., 2019). In the experiment run by Selemani (2015), soil organic matter, nitrate nitrogen, soil organic carbon and exchangeable calcium were both lower by 30–60% when compared to that of enclosures. Schrama et al. (2013) also highlighted that, other factors such as nutrients addition from dungs and urine during grazing can also alter the condition of the soil. Moreover, even on physical properties overgrazing increased topsoil’s temperature and increasing bulk and particle density, which all can be attributed to animal pressure to the rangeland. The increased densities and loss of moisture from the topsoil will make it less suitable for seed germination, thus a decline in species composition will occur and paving way for invasive species (MacLachlan, 2013). However, in enclosures (in rotational grazing) species composition is enriched, less bare areas that will be prone to erosion and leaching away of important soil nutrients vital for plant growth and development (MacLachlan, 2013). Communal property association rangeland users need more knowledge on the state of their respective grazing lands and also the interaction of soil properties with grazing management implemented. Acquiring this in-depth knowledge is of paramount importance to these farmers in developing suitable and sustainable grazing management strategies that will promote livestock production. Research is silent on how grazing management in South African CPAs affect soil productivity, thus this study aimed to explore how grazing management implemented in the CPAs of Bela-Bela affect the soil productivity. It was hypothesized that soils under these CPAs had been negatively affected by grazing in both physical and chemical properties. Therefore, the objective of the study was to assess the effect of grazing management (continued grazing) on the available soil minerals in the four different soil types found in selected CPAs of South Africa.

## Materials & Methods

### Study site description

**Figure 1:**
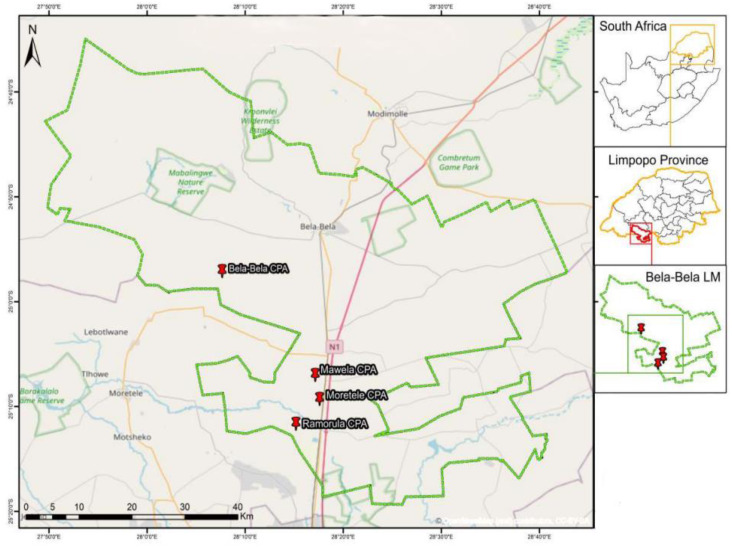
Map location of four selected CPAs.

This study was conducted in Bela-Bela local municipality at four CPAs namely, Mawela, Bela-Bela, Ramorula and Moretele (Table 1) located at the following altitude and coordinates: 1082 m; 25°6′54.30″S, 28°16′52.96″E; 1118 m; 24°57′2.49″S, 28°7′38.03″E, 1036 m; 25°11′36.55″S, 28°14′54.25″E, 1063 m; 25°9′14.78″S, 28°17′34.06″E, respectively. The Bela-Bela local municipality (Fig. 1) is located in the southern part of the Limpopo province. The veld type is springbokvlakte thornveld, open to dense thorn savannah with low shrub layer mainly dominated by acacia species. The geology of the area shows Hutton and Ecca soils with high calcium carbonate content and gilgai micro-relief. Bela-Bela receives an average of 500 to 600 mm of rainfall per year, and the mean daily temperature varies from 5−35 °C throughout the year (Mucina & Rutherford, 2006). The study area is dominated by both some grass and woody species such as *Cymbopogon pospischilii, Aristida cogesta, Cynodon dactylon, Digitaria eriantha, Dichrostachys cinerea* (N-fixer)*, Grewia flava* (N-fixer)*, Senegalia mellifera* (N-fixer) and *Ziziphus mucronata.* The sites were assessed for carrying capacity in order to determine grazing capacity (ha/LSU) following the assessment guideline as per (Van Oudtshoorn, 2015). The following equation was used for grazing capacity: Grazing capacity (ha/LSU): d/(DM/r); d: number of days in a year; DM: dry matter weight in kg (biomass); r: utilisation factor (2.5% of 450 kg body weight). These farms are occupied by cattle, goats, sheep and some game animals. Among the CPAs, Ramarula is the oldest and was obtained in 1998 (24 years ago).

**Table 1:** Profile of all four selected CPA farms in Bela-Bela municipality.

	Mawela	Bela-Bela	Moretele	Ramorula
Year obtained	2008	2007	2003	1998
Farm size	1457 ha	600 ha	2000 ha	850 ha
Soil type	Hutton-clay loam	Hutton-clay	Hutton-loamy sand	Ecca-sand clay loam
Vegetation type	The veld type is springbokvlakte thornveld, open to dense thorn savannah with low shrub layer (Mucina & Rutherford, 2006)			
Altitude and coordinates	(1082 m) 25°6′54.30″S 28°16′52.96″E	(1118 m) 24°57′2.49″S 28°7′38.03″E	(1063 m) 25°9′14.78″S 28°17′34.06″E	(1036 m) 25°11′36.55″S 28°14′54.25″E
Rainfall and temperature	An average of 500 to 600 mm per year, and the mean daily temperature varies from 5–35 °C throughout the year			
Biomass (kg/ha)	658.9	823.3	265.6	488.9
Basal cover (%)	42.39	55.75	28.37	37.22
Grazing capacity (ha/LSU)	7.02	5.82	20.64	8.65

 The lab soil analysis of the study was done at the North-West University experimental farm (Molelwane), Mafikeng (25°47′27″S and 25°37′18″E), North-West province of South Africa, with an altitude of about 1290 m above sea level. All farms were geo-referenced using GPS.

### Soil sampling and analysis

Soil data collection was done between January to February 2021. Topsoil was sampled at a depth of 0 to 150 mm whereas subsoil was sampled from 150 to 300 mm at an interval of 100 m (100–200 m) from the same transect used for woody species data collection resulting in a total of 18 samples per CPA. A 102 mm auger was used when collecting the soil. In each CPA, three camps were selected. In each camp, three transects 200 m apart at the length of 200 m were set. In each transect, soils were drawn at 0,100 and 200 m making a total of nine soil samples per each camp. Sub-samples were bulked per CPA, air-dried, and sieved through a two-millimetre mesh screen pending analysis. Soil pH was analysed as described by McLean (1983), soil organic carbon (OC) was determined by wet oxidation methods of Walkley & Black (1934). Soil (Hutton-clay loam: HCL; Hutton-clay: HC; Hutton-loamy sand: HLS and Ecca sand-clay loam: ESCL) samples from all CPA farms were analyzed for both macro and micro minerals following the guidelines provided by the Agri-Laboratory Association of South Africa (AgriLASA, 1998) and N-NO_3_, N-NH_4_ were determined by the Kjeldahl method. The pH of the soil was determined using a 1:2.5 soil water relation extraction method. Magnesium, Ca, Zn, Cu, Na, Fe, Mn were all determined by atomic absorption spectroscopy, while K was determined by the emission spectroscopy. Ultraviolet spectrophotometer was used to determine P (Olsen & Sommers, 1982). Using an ion chromatograph, the amounts of chloride and sulfate in the soil were determined according to Dick & Tabatabai (1979) and Tabatabai & Dick (1983) methods. Soil texture (particle size) was determined employing the standard Bouyoucos (hydrometer) method (Day, 1965). The soil was also classified according to structure and texture.

### Resistance analysis

Soil resistance measures were used in a field method for measuring soil salinity. The US Bureau electrode cup was filled with soil, which was then moistened with de-ionised water while being stirred with a spatula until a homogenous mixture was obtained. The mixture was consolidated by tapping the container on the workbench from time to time, then testing for the properties of a saturated paste and adding more water if necessary was done (US Salinity Laboratory, 1954). After an hour, it was checked to see if the paste still retained saturated qualities. The sample was allowed to stand for 4 h before determining the electrical resistance of the paste in ohms using a resistance bridge corrected for a temperature of 25 °C. It should be noted that the determination reported by the US Salinity Laboratory corrects the resistance (Ω) to a temperature of 15.5 C.

This study was approved by the North-West University Ethics Committee standards: Ethical Clearance No: NWU-01732-20-A9.

### Statistical analysis

Two-way factorial analysis of variance (SAS, 2010) was used to test the effect of soil depth and soil type in all measured parameters in studied CPA farms. The following model was used for statistical analysis:

\begin{eqnarray*}Yij=\mu +Pi+Sj+(Pi~x~Sj)+ij \end{eqnarray*}

where Yij was the dependent variable (physical and chemical soil properties), µwas the overall mean, *P* was the soil depth effect, *S* was the effect of soil type (different CPAs), and *ɛ* was the random error associated with observation *i j* assumed to be randomly distributed. Statistical difference was acknowledged at *P* < 0.05. Tukey’s test was used to separate the means. *n* = 3 for each subgroup.

## Results

### Soil pH, nitrate-nitrogen, ammonium-nitrogen and organic carbon

**Figure 2:**
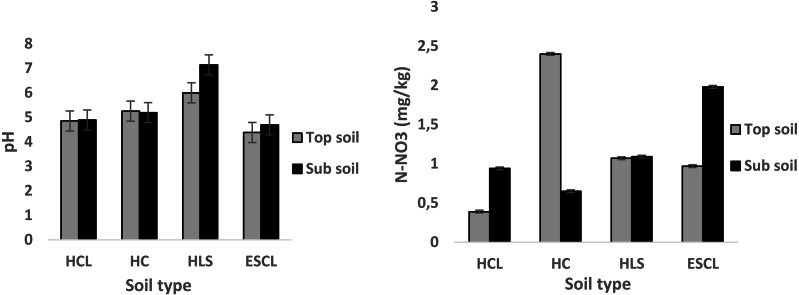
Soil pH and nitrate-nitrogen (mg/kg) found in four soil types of Bela-Bela municipality (*n* = 3). (HCL, Hutton clay-loam; HC, Hutton-clay; HLS, Hutton sandy-loam; ESCL, Ecca sandy clay-loam).

**Figure 3:**
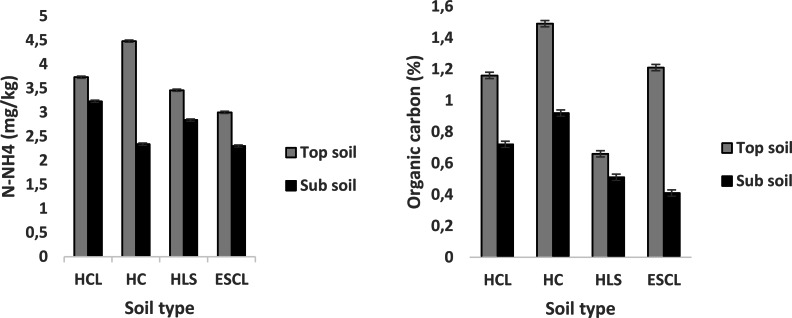
Soil ammonium-nitrogen (mg/kg) and organic carbon (%) found in four soil types of Bela-Bela municipality (*n* = 3). (HCL, Hutton clay-loam; HC, Hutton-clay; HLS, Hutton sandy-loam; ESCL, Ecca sandy clay-loam).

Results on the effect of soil type and depth on soil pH, nitrate-nitrogen (N-NO_3_), ammonium-nitrogen (N-NH_4_) and organic carbon concentration in four different soil types of Bela-Bela municipality are presented in Figs. 2 and 3. There was a statistical significant difference of soil type, depth and the interaction between the two. There was no significant (*P* > 0.05) difference observed in pH of topsoil across the different soil types. The highest (*P* < 0.05) pH (M ±SE: 7.14 ± 0.41) recorded on the sub-soil was in HLS. Nitrate nitrogen (2.4 ± 0.018 mg/kg) concentration on the topsoil was high (*P* < 0.05) in HC soil type when compared to other soil types. In sub-soil, soil nitrate-nitrogen (1.98 ± 0.018 mg/kg) was greater (*P* < 0.05) in HLS soil type compared to other soil types. Soil N-NH_4_ (4.48 ± 0.022 mg/kg) in HC soil type was more (*P* < 0.05) on the top soil than in sub-soil. All soil types had more organic matter on top soil when compared to sub-soil.

### Soil macro minerals

There was a statistical significant difference of soil type, depth and the interaction between the two in the macro minerals in all selected CPAs (Table 2). Calcium concentration was highest (*P* < 0.05) in the topsoil and sub-soil of HC soil type (1420 mg/kg) and HLS soil type (1630 mg/kg), respectively. Phosphorus concentration was significantly high (*P* < 0.05) in ESCL soil type in both the topsoil (12.86 mg/kg) and sub-soil (1.59 mg/kg). A significantly higher (*P* < 0.05) concentration of potassium was observed in topsoil as compared to the sub-soil in ESCL soil type. Sulfate ions concentration was significantly high (*P* < 0.05) in HC soil type (82.9 mg/kg) when compared to other soil types. For both topsoil and sub-soil magnesium concentration was significantly higher (*P* < 0.05) in HC soil type.

### Soil trace/ micro minerals

Results on the effect of soil type and depth on micro mineral concentration in four different soil types of Bela-Bela municipality are presented in Table 3. There was a statistical significant difference of soil type, depth and the interaction between the two. Iron concentration in both topsoil (11.8 mg/kg) and sub-soil (7.3 mg/kg) was significantly high (*P* < 0.05) in ESCL soil type when compared to other soil types. In sub-soil, manganese concentration was found to be higher (*P* < 0.05) in ESCL soil type (7.58 mg/kg). Copper concentration was high (*P* < 0.05) in HC soil type for both the topsoil (2.61 mg/kg) and sub-soil (3.54 mg/kg). The highest (*P* < 0.05) concentration of chlorine (42.2 mg/kg) was observed in the topsoil of ESCL soil type, whereas in the subsoil the highest (*P* < 0.05) concentration (66 mg/kg) was found in HCL soil type.

**Table 2:** Macro mineral (mg/kg) found in four different soil types of Bela-Bela municipality (*n* = 3).

	Ca		P		K		Na		SO_4_		Mg	
Soil type	Top	Sub	Top	Sub	Top	Sub	Top	Sub	Top	Sub	Top	Sub
HLC	679.0^cA^	618.0^cB^	2.57^dA^	0.32^cB^	221.0^bA^	144.0^bA^	5.78^cB^	7.84^bA^	35.3^cB^	85.9^aA^	288.0^bA^	288.0^bA^
HC	1420.0^aB^	1340.0^bA^	8.74^bA^	0.14^dB^	394.0^aA^	302.0^aB^	7.39^bA^	7.39^cA^	82.9^aA^	76.2^bA^	480.0^aA^	359.0^aB^
HLS	845.0^bB^	1630.0^aA^	5.21^cA^	1.12^bB^	120.0^cA^	149.0^bB^	4.95^dB^	7.21^cA^	32.2^cB^	84.5^aA^	98.0^dB^	138.0^dA^
ESCL	333.0^dB^	353.0^dA^	12.86^aA^	1.59^aB^	125.0^cA^	109.0^cB^	8.49^aB^	14.90^aA^	72.9^bB^	41.6^cA^	129.0^cB^	156.0^cA^
S.E	2.28		0.018		1.72		0.074		1.08		2.65	

**Notes.**

a,b,c,d Means in the same column, with different superscripts are significantly different (*P* < 0.05).

AB Means with different superscripts within each soil type are significantly different (*P* < 0.05).

Ca Calcium P Phosphorus K Potassium Na Sodium SO_4_ Sulfate Mg Magnesium HCL Hutton-clay loam HC Hutton-clay HLS Hutton-loamy sand ESCL Ecca sandy-clay loam SE Standard error

**Table 3:** Micro minerals (mg/kg) found in four soil types of Bela-Bela Municipality (*n* = 3).

	Fe		Mn		Cu		Cl		Zn	
Soil type	Top	Sub	Top	Sub	Top	Sub	Top	Sub	Top	Sub
HCL	5.22^bA^	3.37^cB^	22.60^bA^	4.34^cB^	2.03^bB^	2.26^bA^	18.0^bcB^	66.0^aA^	1.83^aA^	0.44^bB^
HC	5.43^bA^	3.90^bB^	40.80^aA^	7.27^abB^	2.61^aB^	3.54^aA^	41.0^aA^	41.2^bA^	3.28^aA^	1.37^aB^
HLS	2.09^cA^	1.48^dB^	14.60^cA^	6.23^bB^	0.31^dB^	0.43^dA^	16.0^cB^	41.1^bA^	0.94^dA^	0.44^bB^
ESCL	11.80^aA^	7.30^aB^	21.50^bA^	7.58^aB^	0.74^cA^	0.79^cA^	42.2^aA^	26.2^cB^	1.10^cA^	0.42^bB^
S.E	0.078		0.23		0.016		0.90		0.097	

**Notes.**

a,b,c,d Means in the same column, with different superscripts are significantly different (*P* < 0.05).

AB Means with different superscripts within each soil type are significantly different (*P* < 0.05).

Fe Iron Mn Manganese Cu Copper CL Chlorine Zn Zinc HCL Hutton-clay loam HC Hutton-clay HLS Hutton-loamy sand ESCL Ecca sandy-clay loam SE Standard error

### Particle size distribution of different soil types

Results of the particle size distribution of sand, clay, and silt found in four different soil types and depth of Bela-Bela municipality are presented in Table 4. There was a statistical significant difference of soil type, depth and the interaction between the two. Sand particles (84%) on the topsoil were more (*P* < 0.05) distributed in HLS soil type compared to other soil types. There was a significant (*P* < 0.05) increase in clay particle size (24%) distribution in topsoil of HC soil type compared to other soil types. In sub-soil, the highest (*P* < 0.05) distribution of clay particles (30%) was observed in HC soil compared to other soil types. For both the topsoil (0.14) and sub-soil (0.09), the acidity was significantly high (*P* < 0.05) in the ESCL soil type.

**Table 4:** Soil particle size distribution of sand, silt, clay (%), and acidity found in four different soil types (*n* = 3).

	Sand		Silt		Clay		Acidity	
Soil type	Top	Sub	Top	Sub	Top	Sub	Top	Sub
HCL	72.0^abA^	68.0^aA^	6.0^aA^	8.0^aA^	22.0^aA^	24.0^abA^	0.03^bA^	0.03^bA^
HC	68.0^bA^	60.0^bA^	8.0^aA^	10.0^aA^	24.0^aA^	30.0^aA^	0.02^cA^	0.02^cA^
HLS	84.0^aA^	80.0^aA^	4.0^aA^	6.0^aA^	12.0^bA^	14.0^cA^	0.01^dB^	0.04^bA^
ESCL	76.0^abA^	76.0^aA^	4.0^aA^	4.0^aA^	20.0^abA^	20.0^bcA^	0.14^aA^	0.09^aB^
S.E	2.72		1.31		1.97		0.0016	

**Notes.**

a,b,c,d Means in the same column, with different superscripts are significantly different (*P* < 0.05).

AB Means with different superscripts within each soil type are significantly different (*P* < 0.05).

HCL Hutton- clay loam HC Hutton- clay HLS Hutton- loamy sand ESCL Ecca sandy-clay loam SE Standard error

### Resistance of four different soil types

**Figure 4:**
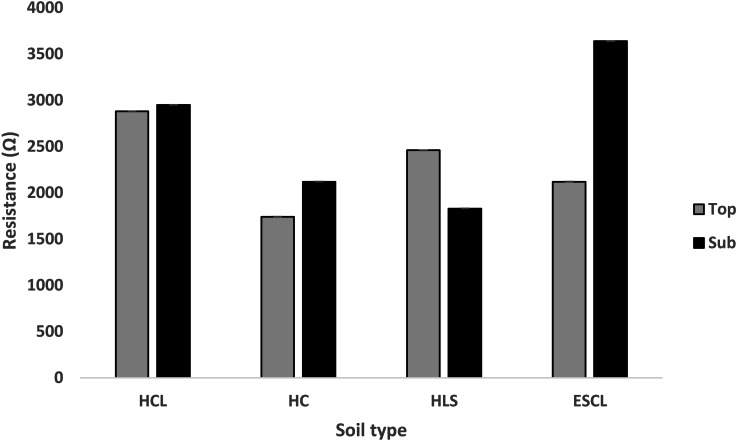
Mean values for resistance (Ω) of four different soil types in the Bela-Bela Municipality (*n* = 3). (HCL, Hutton clay-loam; HC, Hutton-clay; HLS, Hutton sandy-loam; ESCL, Ecca sandy clay-loam).

Figure 4 depicts the mean resistance of four different soil types and depth of Bela-Bela municipality CPAs. There was a statistical significant difference of soil type, depth and the interaction between the two. Soil resistance (M ±SE: 2880 ± 1.67 Ω) measured in topsoil was high (*P* < 0.05) in HCL soil type compared to other soil types. Moreover, for the sub-soil, the highest (*P* < 0.05) resistance (3640 ± 1.67 Ω) was recorded in ESCL soil type.

## Discussion

### Soil pH, nitrate-nitrogen, ammonium nitrogen and organic carbon

Grazing alters both physical and chemical properties of the soil by removing biomass and trampling it (Wang & Wesche, 2016a; Wang & Wesche, 2016b). Moreover, pH under uncontrolled or heavy grazing tends to rise (Steffens et al., 2008; Wang & Wesche, 2016a; Wang & Wesche, 2016b). The acidity and alkalinity of a soil solution are measured by its pH, which is altered by both acid and base-forming ions in the soil (McCauley, Jones & Jacobsen, 2009). Furthermore, Minasny et al. (2016) and Neina (2019) highlighted that the pH of the soil does indeed have a huge impact on soil biological processes in the natural environment. As a result, soil pH is referred to as the “chief soil factor”, influencing a wide range of biological, chemical, and physical processes that affect plant development and vegetative growth. In the current study, the highest pH (7.14) was recorded in the HLS soil type. As per the explanation by Lauber et al. (2009) and Tripathi et al. (2012), pH around neutral to alkaline indicates richness in the bacterial community in the soil as they thrive well in these pH ranges. Furthermore, the observed highest pH reading (7.14) is within the range reported by Tripathi et al. (2012) who studied forest and open-land sites. pH either positively or negatively affects plant growth, Macdonald et al. (2014) noted that plant growth is limited in acidic soils. The low productivity of such soil points out the need of finding a better way to alleviate such constraints in plant production under acidic soils. Studies by Raiesi & Riahi (2014) and Ebrahimi, Khosravi & Rigi (2016) conducted on natural over grazed rangelands revealed that the presence of livestock increased soil pH essentially by the hydrolysis of urine urea in the grazing area, hence the current study pH result can also be attributed to the continuous grazing practiced in these farms.

Acidic soils also limit plants from accessing very important nutrients for development such as Mg, N, P, Mn and S (Macdonald et al., 2014), instead, these soils promote the availability of potentially toxic (Al and Bo) nutrients when in excess (Zhao, Chen & Shen, 2014). Land degradation is a worldwide problem for rangeland ecologists, and it is caused by factors such as long-term drought and erosion, but overgrazing is deemed the principal contributing factor to rangeland degradation. Studies by Li et al. (2014) and Raiesi & Riahi (2014) revealed that degraded lands have high pH values, as shown by a shift in pH values from a pre-degradation state (8.7) to a post-degradation state (9.42). Both acidity and alkalinity can promote toxicity, high pH increases osmotic stress and ion toxicity (Shi & Wang, 2005).

Nitrogen is an essential nutrient that is found in the largest proportion of plants and can sometimes hinder plant development and yield. Nitrogen is found quite often in soils as a portion of organic matter (Miller & Sonon, 2014). Abril & Bucher (1999), noted that soil compaction, reduced water retention, increased salinity, and the loss of certain soil nutrients, especially nitrogen, are all consequences of overgrazing. Even though the highest nitrate-nitrogen in the present study was recorded in the HC soil type, but still, fell below 10 mg/kg (Waller & Kookana, 2009), the recommended minimum level for plant growth. In a study by Šimek & Cooper (2002), it was revealed that denitrification was quick in soils with a pH greater than 5.8 than those with a pH range of 3.6 to 4.1. Indeed, the low amount of nitrate-nitrogen than the standard recommendation for plant growth might be attributed to denitrification taking place in these soils.

Chartier, Rostagno & Pazos (2011) reported that low content of nitrogen, nitrate and ammonium are characteristics of degraded rangelands due to prolonged overgrazing. Furthurmore, Bisigato, Laphitz & Carrera (2008) and Schiettecatte et al. (2008) also alluded that soil nitrogen is vastly correlated to carbon. Visual observation and farmers’ responses during data collection confirms that these farms have been heavily grazed in the past 10 years. Contrary to what was expected and obtained in the current study, Northup, Starks & Turner (2019a, 2019b) who studied the USDA-ARS grazing lands at Oklahoma reported that nitrogen increased with continuous and high stocking rates, especially near watering points and some corners of occupied paddocks. Ammonium (NH4^+^) fixation and release can also have a significant impact on the availability of nitrogen (Steffens & Sparks, 1999; Juang et al., 2001) because it instigates the available nitrogen (N) for uptake by the plants after defixation has taken place. In the current study, the concentration of ammonium-nitrogen was observed to be less in topsoil than in subsoil, these can be attributed to the high demand of N by plants when recovering in response to defoliation, hence ammonium fixation takes place to make N available to plant. As Liu et al. (2008) alluded that an increase in the fixation of NH4^+^ does help build up the accessible amount of N in the soil, allowing plants to recover rapidly and halting the loss of nitrogen to the environment.

Livestock has a vital role to play in soil properties through trampling that increase the bulkiness (Rapti et al., 2016). Moreover, Noellemeyer, Quiroga & Estelrich (2006) highlighted that heavy grazing, on the other hand, can lead to rangeland degradation which will drastically decrease the herbaceous vegetation and further instigate soil erosion. Soil is indeed the biggest terrestrial carbon and nitrogen storehouse, soil can store three times much more carbon and nitrogen than the atmosphere (Stuart Chapin III et al., 2009), primarily in the form of decayed plant litter and residues (Lal, 2004; Yusuf, Treydte & Sauerborn, 2015). Several researchers have highlighted that even grazing does influence the available organic carbon in the soil (Reeder & Schuman, 2002; Shrestha & Stahl, 2008; Yusuf, Treydte & Sauerborn, 2015).

Grazing by livestock does affect soil organic carbon, overgrazing is considered to reduce soil carbon and nitrogen by the removal of the aboveground herbaceous vegetation cover directly from the soil, reducing potential carbon dioxide fixation in photosynthetic plants tissues. Furthermore, carbon dioxide belowground will be reduced through the shortfalls of root development and higher root litter turnover (Reeder et al., 2004; Semmartin, Di Bella & De Salamone, 2010).

Soil organic carbon (SOC) (0.41–1.21) in this study is lower than the reported range (1.43–2.1%) by Yusuf, Treydte & Sauerborn (2015), Abule, Smit & Snyman (2005), and Belay & Kebede (2010) who all conducted studies in semi-arid rangelands. Wang et al. (2018) reported higher (6.92%) SOC than the present study, and it was noted that the variation amongst SOC was caused by particle size distribution, altitude and rainfall as the main contributors in semi-arid rangelands. Subsequently, Somenahally et al. (2020), also reported 1.6% and 25.1% of soil organic carbon for both top and subsoil respectively under high grazing pressure, these results are higher than those obtained in the current study. The variation in the reported organic carbon could be explained by the different woody species encroaching and grazing management employed by these farms. Another key restriction on the decomposition rate is the quality of carbon sources, which are generally determined by their lignin content (Jobbágy & Jackson, 2000). Woody species are said to have high lignin content than grasses, due to (their woody plants) being slowly decomposable can increase the storage of SOC. Woody species might contribute more to the SOC content, but also dead grass biomass does increase SOC (Dinakaran, Mehta & Krishnayya, 2011). Indeed, the current results agree on the relationship between HC soil type biomass and the SOC content found in the same soil type. Moreover, good basal cover and high biomass of the rangeland will promote more storage of SOC (Shiferaw, Yimer & Tuffa, 2019).

### Macro and micro minerals

The presence of macronutrients, as well as the amount in which they are available, plays a huge role in plant development, plant vigor and yield (Northup, Starks & Turner, 2019a and Northup, Starks & Turner, 2019b). According to Baron et al. (2001), generally, every grazing management strategy employed will have an impact on the nutrient cycle and the net nutrient reservoirs throughout the soil. In the current study, there was a significant difference observed amongst macronutrients across all soil types. Chlorine was significantly higher in both top and subsoil in ESCL and HCL respectively. Hook & Burke (2000); Briske, Fuhlendorf & Smeins (2005); Northup, Starks & Turner (2019a) and Northup, Starks & Turner (2019b) stressed that the type of plant community, location within the landscape, and temperature all influence macronutrients abundance and distribution. These characteristics tend to produce varied distributions that cannot be traced back to grazing impacts (Oades, 1993; Northup, Starks & Turner, 2019a; Northup, Starks & Turner, 2019b). As noted by Neina (2019) plants can access some nutrients at different pH values, for instance, magnesium and calcium their highest value (top soil and sub soil) were recorded in both HC (pH 5.25) and HCL (pH 4.89) soil types. Moreover, Penn & Camberato (2019) noted that phosphorus (P) solubility normally occurs around the pH of 4.5 to 6.5. However, although the exact P solubility will vary in terms of soil type, geographical location, and climate, the range close to neutral is the most sound, as it is regarded as the best pH range for optimum plant growth. This is comparable to the assertion by McCauley, Jones & Jacobsen (2009), that these macronutrients are most accessible to plants at pH range of 6 to 8. The good basal cover in the HC soil type might be due to the nutrients being available to the plants accompanied by good grazing management employed in this soil type. However, inconsistencies of soil nutrients in these soil types might be caused by uncontrolled or heavy grazing practiced in these farms. Large quantities of mineralization increased nutrient uptake by stressed plants (Crovo et al., 2021; Abdalla et al., 2018), and this can play part in lowering the available macro nutrients in the soil. Moreover, Ragimov et al. (2020) also alluded that high grazing intensity does decline in the net productivity of the rangeland, resulting in the defeat of palatable species and soil chemical properties. Contrary to the aforementioned, Rutherford & Powrie (2010) and Al-Rowaily, El-Bana & Al-Dujain (2012) found that short-term grazing improved the concentration of some of soil minerals such as SOC, N, K and P in a depth of 0–30 cm.

The current study recorded the lowest pH values in both HCL and ESCL soil types, meaning that these soils are acidic. Acidic soils (with pH <5.5), due to different contributing factors are known to hinder plant growth and the toxicity of aluminium (Al) is thought to be the principal limiting factor of plant growth in acidic soils (Kochian, Pineros & Hoekenga, 2005; Zhao, Chen & Shen, 2014). To counteract such a problem in plant production, in plants under Al stress, the availability of phosphorus (P) and ammonium has been noted to ameliorate Al toxicity and diminishes the release of organic acids from plant roots (Sun et al., 2008; Chen, Zhao & Shen, 2010; Zhao, Chen & Shen, 2014). This suggests that if both HCL and ESCL soil types were to accumulate more of both Al and P that would aid in increased toxicity and the betterment of the soil’s pH respectively.

Variation in phosphorus (P) concentration obtained from ESCL soil type might be explained by the time (summer) the soil samples were collected. During, the growing season plants when grazed continuously due to mismanagement, respond by up taking more minerals from the soil and the results from the current study revealed lower P concentration in the subsoil than that of topsoil. Complementary to the abovementioned, the same was observed with potassium, as it was more on the topsoil than in subsoil. The vegetation layer especially herbs (Fujita et al., 2010) might have taken much of the top soil P and K which lead to reduced concentration of these minerals in the top soil (Van der Salm et al., 2012). Moreover, environmental conditions such as runoff and erosion might have contributed to the leaching of these soil minerals (Andersson et al., 2013), and these can normally take place in overgrazed areas. Sulfur (S) is consistently being cycled amid inorganic and organic forms in soil and the release of SO_4_ from organic S forms is particularly of colossal significance to plants (Kertesz & Mirleau, 2004; Wilhelm, 2009). In the current study, sulfate ions were significantly high in both HC and HCL soil types. The high levels of sulfate ions in the two soil types might be attributed to sulphur that is deposited on the ground as urine and dung from livestock. As explained by Whitehead (2000); Northup, Starks & Turner (2019a) and Northup, Starks & Turner (2019b), livestock retains around 25% of the S in the forage they consume, with the rest eliminated from the body as dung and urine. Sulfur availability to plants can be achieved by making a couple of water points in the paddocks where livestock graze, as it has been reported that sulphur concentration tends to increase close to water points (Haynes & Williams, 1993). The proper accumulation of macro minerals in the soil will sure elevate biomass and vegetation cover when proper grazing management is deployed.

Micronutrients are commonly regarded as important plant nutrients that are taken by plants from the soil in fewer quantities and they play a vital role in plant growth, plant metabolism, and development (Tavakoli et al., 2014). Furthermore, with them being deficient, plants can be easily attacked by diseases (Monreal et al., 2016). Concerning micronutrients, there was a significant variation across soil types for both topsoil and subsoil. According to Shrivastav et al. (2020), the majority of micronutrients such as copper (Cu), zinc (Zn), and iron (Fe) are mostly available to plants within the pH range of five to seven. Any variation from the ideal range of this pH will slow and reduce nutrient availability, making them less accessible to plants. The current study results were lower when compared to those reported by Márquez-Madrid et al. (2017) who conducted a study in grassland sites of Zacatecas, this might be attributed to the low pH range of the above-mentioned one and also pH varies with location, soil texture and soil structure. (Goulding, 2016). Bradl (2004) and Neina (2019) alluded that at low pH micronutrients are normally soluble due to high desorption and low adsorption. Under certain assumptions, this can be construed as the reason behind the low content of micronutrients in the soil, plants might have been responding to grazing by livestock.

Manganese (Mn) is one of the trace elements that are required for plant growth. It is indeed partaking in some enzymatic reaction that helps in photosynthesis (Michopoulos et al., 2021). Concerning, manganese concentration in the current study, it was recorded to be high in topsoil than in subsoil in ESCL soil type. The low concentration of Mn in subsoil might be attributed to the uptake by plants (Michopoulos et al., 2021), as the low pH enhances the Mn uptake and the pH range of this soil type is suitable for Mn to be more available to plants. Furthuremore, Gandois & Probst (2012) (373 mg/kg) and Michopoulos et al. (2021) (95.4–148 mg/kg) both reported a high concentration of soil Mn when compared to the present study. The fair basal cover in ESCL soil type might have been influenced by the Mn uptake.

### Soil particle size, acidity and resistance

According to Su et al. (2004a, 2004b) soil is classified in different ways and particle size distribution (PSD) is the most frequently used technique to estimate a lot of soil-related properties. PSD has a huge impact on how water, ion movements, heat and air movement are retained in the soil. Losses of soil minerals such as organic carbon and other minerals will cause a decline in the water holding capacity, altering soil structure as well as some biotic qualities which are all coupled by partial dissolution of small particle size fractions in rangeland soils (Su et al., 2004a; Su et al., 2004b; Bronick & Lal, 2005). Particle size distribution play a vital role in agricultural land productivity. In addition, Amakor et al. (2014) and Mandal et al. (2015) alluded that to manage land resources sustainably, accurate and exact salinity measures are essential and need to be accessed, particularly in terms of soil quality and rangeland productivity. Hutton and Ecca soil types contained clay and sandy-loam content in them (Mengistu, Mavimbela & Van Rensburg, 2019). It has been reported by Bennie & Hensley (2001) and Mengistu, Mavimbela & Van Rensburg (2019) that clay and silt fine particles in the sandy soils play a major role in the drainage and water holding capacity. Moreover, as displayed in Table 4, the most dominant PSD across all soil types was sand particles with clay particles coming in second. This suggests that these tiny sand particles can be easily removed by the wind in grazing lands that have large bare areas, slowly causing the rangelands to be degraded with time. However, according to Parwada & Van Tol (2017), clay material provides the necessary bonding between the various soil particles (sand, silt and clay), bringing about the production of more stable aggregates that are less prone to erosion. Species (both grasses and browse) are adapted to different habitats. The high presence of sand particles in HLS soil type allowed the dominance of grasses and woody species such as *Cynodon dactylon*, *Digitaria eriantha*; *Grewia flava* and *Senegalia mellifera* (Van Wyk, Van Wyk & Van Wyk, 2012; Van Oudtshoorn, 2020) as these species were noted to be present in the study area. While on the other hand clay particles in the HCL soil type permitted the growth of species such as *Cymbopogon pospischilii*, *Aristida cogesta*, *Dichrotachys cinerea*, and *Grewia flava* as mentioned by the same authors (Van Wyk, Van Wyk & Van Wyk, 2012; Van Oudtshoorn, 2020).

Land deterioration owing to soil acidity is one element of land degradation that limits rangeland productivity worldwide (Abate et al., 2017). In addition, soil acidity is caused by land degradation owing to overgrazing, deforestation, and continuous cultivation (Dejene, 2003; Taddese, 2001). The pH is a parameter used to measure soil acidity, in agreement with the current study pH readings in ESCL soil type recorded the most acidity in the soil. The current study results of acidity are low, and as highlighted by Abate et al. (2017), acid soils are predominant in high rainfall areas as compared to those with low rainfall. At this stage of understanding, one would not expect acid soils in these farms where the current study was done.

The electrical resistance of a saturated soil paste is a function of the soil salt concentration and is inversely proportional to salt concentration (US Salinity Laboratory, 1954). Ideally, the most commonly used method for determining salinity is to test electrical conductivity in saturated paste extracts. In the current study soils HCL, HC and ESCL their resistance in the subsoil is higher than in the topsoil meaning there is less salts in subsoil than topsoil. Whereas soil HLS resistance in the topsoil is higher than in the subsoil meaning there is less salts in the topsoil than in the subsoil. Continuous grazing does encourage salinization in soils (Chaneton & Lavado, 1996), which also promotes high temperatures and evaporation. In agreement with the with the current study results, Chaneton & Lavado (1996) and Sepehry et al. (2012) found that the top soil in continuously grazed camps had high salinity levels when compared to lightly grazed camps. With that being said, such soils in continuously grazed rangelands will retain a lot of salts (Van Rensburg et al., 2011) if there is not much rainfall to leach them out, thus decreasing the resistivity. The resistance obtained in the study is high, giving insight or suggesting that the soils in the current study have low salts concentration. Furthermore, the high levels of soluble salts will surely hinder the growth of salt sensitive plants leading to reduced plant yields in these soil types.

## Conclusions

The objective of the study was to assess how grazing management affected the available soil mineral of these four different soil types found in the Bela-Bela municipality. Grazing management in these selected CPAs is critical for maintaining rangeland productivity and sustainability as grazing by livestock plays a vital role in the nutrient cycle in the soil. Hutton loamy-sand soil type had an intermediate pH that permits the availability of most macro and micronutrients. This suggests that this soil type would perform well given a good grazing management strategy is used making soil nutrients available to plants thus eventually increasing the basal cover and biomass production. All soil types had more concentration of soil nutrients on the topsoil than subsoil. Both macro- and micronutrients analysed from the soil type, nitrogen (N), chlorine (Cl), iron (Fe) and copper (Cu) were all found to be deficient to support plant growth, as they were below 6.4 g/kg (Hengl et al., 2021). Given the deficiency of nitrogen in the soils, farmers in these CPAs should prioritize more enclosures and keep some leguminous plants as they will aid in restoring N content. As far as acidity is concerned, all these soil types are not acidic, as this was expected because these soil types (CPA farms) are not situated in high rainfall areas. From the above results, emerging key findings boils back to the type of grazing management employed in these farms as it accounts for a wide range of things happening in the soil from basal cover to erosion which will lead to poor soil. In short, improved grazing management will surely positively affect the rangeland productivity, health, and sustainability.

##  Supplemental Information

10.7717/peerj.13960/supp-1 Supplemental Information 1 Data on pH, P-Bray, Resistance, Organic Carbon and Acidity of top-soil found in four selected CPAs in Bela-Bela municipality, South Africa

10.7717/peerj.13960/supp-2 Supplemental Information 2 Data on pH, P-Bray, Resistance, Organic Carbon and Acidity of sub-soil found in four selected CPAs in Bela-Bela municipality, South Africa

10.7717/peerj.13960/supp-3 Supplemental Information 3 Data on soil particle size of top-soil found in four selected CPAs in Bela-Bela municipality, South Africa

10.7717/peerj.13960/supp-4 Supplemental Information 4 Data on soil particle size of sub-soil found in four selected CPAs in Bela-Bela municipality, South Africa

10.7717/peerj.13960/supp-5 Supplemental Information 5 Data on mineral elements of top-soil found in four selected CPAs in Bela-Bela municipality, South Africa

10.7717/peerj.13960/supp-6 Supplemental Information 6 Data on mineral elements of sub-soil found in four selected CPAs in Bela-Bela municipality, South Africa

## Additional Information and Declarations

### Data Availability

The following information was supplied regarding data availability: The raw data are available in the Supplementary Files.

## References

[ref-1] Abate E, Hussein S, Laing M, Mengistu F (2017). Soil acidity under multiple land-uses: assessment of perceived causes and indicators, and nutrient dynamics in small-holders’ mixed-farming system of northwest Ethiopia. Acta Agriculturae ScandInavica, Section B—Soil & Plant Science.

[ref-2] Abdalla M, Hastings A, Chadwick DR, Jones DL, Evans CD, Jones MB, Rees RM, Smith P (2018). Critical review of the impacts of grazing intensity on soil organic carbon storage and other soil quality indicators in extensively managed grasslands. Agriculture, Ecosystems & Environment.

[ref-3] Abril A, Bucher EH (1999). The effects of overgrazing on soil microbial community and fertility in the Chaco dry savannas of Argentina. Applied Soil Ecology.

[ref-4] Abule E, Smit GN, Snyman HA (2005). The influence of woody plants and livestock grazing on grass species composition, yield and soil nutrients in the Middle Awash Valley of Ethiopia. Journal of Arid Environments.

[ref-5] Agrawal A (2001). Common property institutions and sustainable governance of resources. World Development.

[ref-6] AgriLASA (1998). Feed and plant analysis methods.

[ref-7] Al-Rowaily SL, El-Bana MI, Al-Dujain FA (2012). Changes in vegetation composition and diversity in relation to morphometry, soil and grazing on a hyper-arid watershed in the central Saudi Arabia. Catena.

[ref-8] Amakor XN, Jacobson AR, Cardon GE, Hawks A (2014). A comparison of salinity measurement methods based on soil saturated pastes. Geoderma.

[ref-9] Andersson H, Bergström L, Djodjic F, Ulén B, Kirchmann H (2013). Topsoil and subsoil properties influence phosphorus leaching from four agricultural soils. Journal of Environmental Quality.

[ref-10] Ash AJ, Corfield JP, McIvor JG, Ksiksi TS (2011). Grazing management in tropical savannas: utilization and rest strategies to manipulate rangeland condition. Rangeland Ecology & Management.

[ref-11] Ash AJ, Smith DMS, Abel N, Reynolds JF, Stafford Smith DM (2002). Land degradation and secondary production in semi-arid and arid grazing systems. Global desertification: do humans cause deserts?.

[ref-12] Balestrini R, Lumini E, Borriello R, Bianciotto V (2015). Plant-soil biota interactions (Chapter 11). Soil Microbiology, Ecology and Biochemistry.

[ref-13] Baron VS, Dick AC, Mapfumo E, Malhi SS, Naeth MA, Chanasyk DS (2001). Grazing impacts on soil nitrogen and phosphorus under Parkland pastures. Journal of Range Management.

[ref-14] Belay L, Kebede F (2010). The impact of woody plants encroachment on soil organic carbon and total nitrogen stocks in Yabello District, Borana Zone, Southern Ethiopia. Journal of Dryland.

[ref-15] Bennie ATP, Hensley M (2001). Maximizing precipitation utilization in dryland agriculture in South Africa—a review. Journal of Hydrology.

[ref-16] Bisigato AJ, Laphitz RML, Carrera AL (2008). Non-linear relationships between grazing pressure and conservation of soil resources in Patagonian Monte shrublands. Journal of Arid Environments.

[ref-17] Bolo PO, Sommer R, Kihara J, Kinyua M, Nyawira S, Notenbaert AMO (2019). Rangeland degradation: causes, consequences, monitoring techniques and remedies. Working Paper. CIAT Publication No. 478.

[ref-18] Bradl HB (2004). Adsorption of heavy metal ions on soils and soils constituents. Journal of Colloid and Interface Science.

[ref-19] Briske DD, Fuhlendorf SD, Smeins FE (2005). State-and-transition models, thresholds, and rangeland health: a synthesis of ecological concepts and perspectives. Rangeland Ecology and Management.

[ref-20] Bronick CJ, Lal R (2005). Soil structure and management: a review. Geoderma.

[ref-21] Cao J, Xu X, Deo RC, Holden NM, Adamowski JF, Gong Y, Feng Q, Yang S, Li M, Zhou J, Zhang J (2018). Multi-household grazing management pattern maintains better soil fertility. Agronomy for Sustainable Development.

[ref-22] Chaneton EJ, Lavado RS (1996). Soil nutrients and salinity after long-term grazing exclusion in a flooding Pampa grassland. Rangeland Ecology & Management/Journal of Range Management Archives.

[ref-23] Chartier MP, Rostagno CM, Pazos GE (2011). Effects of soil degradation on infiltration rates in grazed semiarid rangelands of northeastern Patagonia, Argentina. Journal of Arid Environments.

[ref-24] Chen ZC, Zhao XQ, Shen RF (2010). The alleviating effect of ammonium on aluminum toxicity in Lespedeza bicolor results in decreased aluminum-induced malate secretion from roots compared with nitrate. Plant and Soil.

[ref-25] Crovo O, Aburto F, da Costa-Reidel C, Montecino F, Rodríguez R (2021). Effects of livestock grazing on soil health and recovery of a degraded Andean Araucaria forest. Land Degradation & Development.

[ref-26] Day PR, Black CA (1965). Particle fractionation and particle-size analysis. Methods of soil analysis. Part 1: Physical and mineralogical properties, including statistics of measurement and sampling.

[ref-27] Dejene A (2003). Integrated natural resources management to enhance food security. The case for community-based approaches in Ethiopia. Working Paper (FAO).

[ref-28] Dick WA, Tabatabai MA (1979). Ion chromatographic determination of sulfate and nitrate in soils. Soil Science Society of America Journal.

[ref-29] Dinakaran J, Mehta N, Krishnayya NSR (2011). Soil organic carbon dynamics in two functional types of ground cover (grasses and herbaceous) in the tropics. Current Science.

[ref-30] Dougill AJ, Heathwaite AL, Thomas DS (1998). Soil water movement and nutrient cycling in semi-arid rangeland: vegetation change and system resilience. Hydrological Processes.

[ref-31] Ebrahimi M, Khosravi H, Rigi M (2016). Short-term grazing exclusion from heavy livestock rangelands affects vegetation cover and soil properties in natural ecosystems of southeastern Iran. Ecological Engineering.

[ref-32] Egeru A, Wasonga O, Gabiri G, MacOpiyo L, Mburu J, Mwanjalolo Majaliwa JG (2019). Land cover and soil properties influence on forage quantity in a semiarid region in East Africa. Applied and Environmental Soil Science.

[ref-33] Fujita Y, Robroek BJ, De Ruiter PC, Heil GW, Wassen MJ (2010). Increased N affects P uptake of eight grassland species: the role of root surface phosphatase activity. Oikos.

[ref-34] Gandois L, Probst A (2012). Localisation and mobility of trace metal in silver fir needles. Chemosphere.

[ref-35] Ghate R, Nagendra H (2005). Role of monitoring in institutional performance: forest management in Maharashtra, India. Conservation and Society.

[ref-36] Goulding KWT (2016). Soil acidification and the importance of liming agricultural soils with particular reference to the United Kingdom. Soil Use and Management.

[ref-37] Haynes RJ, Williams PH (1993). Nutrient cycling and soil fertility in the grazed pasture ecosystem. Advances in Agronomy.

[ref-38] Hengl T, Miller MA, Križan J, Shepherd KD, Sila A, Kilibarda M, Antonijević O, Glušica L, Dobermann A, Haefele SM, McGrath SP (2021). African soil properties and nutrients mapped at 30 m spatial resolution using two-scale ensemble machine learning. Scientific Reports.

[ref-39] Herrick JE, Wander MM (2018). Relationships between soil organic carbon and soil quality in cropped and rangeland soils: the importance of distribution, composition, and soil biological activity. Soil processes and the carbon cycle.

[ref-40] Holecheck J, Galt D, Joseph J, Navarro J, Kumalo G, Molinar F, Thomas M (2003). Moderate and light cattle grazing effects on Chihuahuan Desert rangelands. Rangeland Ecology & Management/Journal of Range Management Archives.

[ref-41] Hook PB, Burke IC (2000). Biogeochemistry in a shortgrass landscape: control by topography, soil texture, and microclimate. Ecology.

[ref-42] Jobbágy EG, Jackson RB (2000). The vertical distribution of soil organic carbon and its relation to climate and vegetation. Ecological Applications.

[ref-43] Juang TC, Wang MK, Chen HJ, Tan CC (2001). Ammonium fixation by surface soils and clays. Soil Science.

[ref-44] Kertesz MA, Mirleau P (2004). The role of soil microbes in plant sulphur nutrition. Journal of Experimental Botany.

[ref-45] Kochian LV, Pineros MA, Hoekenga OA (2005). The physiology, genetics and molecular biology of plant aluminum resistance and toxicity. Plant and Soil.

[ref-46] Lal R (2004). Soil carbon sequestration impacts on global climate change and food security. Science.

[ref-47] Lauber CL, Hamady M, Knight R, Fierer N (2009). Pyrosequencing-based assessment of soil pH as a predictor of soil bacterial community structure at the continental scale. Applied and Environmental Microbiology.

[ref-48] Lavado RS, Sierra JO, Hashimoto PN (1996). Impact of grazing on soil nutrients in a Pampean grassland. Journal of Range Management.

[ref-49] Li Q, Zhou D, Jin Y, Wang M, Song Y, Li G (2014). Effects of fencing on vegetation and soil restoration in a degraded alkaline grassland in northeast China. Journal of Arid Land.

[ref-50] Liu YL, Zhang B, Li CL, Hu F, Velde B (2008). Long-term fertilization influences on clay mineral composition and ammonium adsorption in a rice paddy soil. Soil Science Society of America Journal.

[ref-51] Macdonald LM, Farrell M, Zwieten LV, Krull ES (2014). Plant growth responses to biochar addition: an Australian soils perspective. Biology and Fertility of Soils.

[ref-52] MacLachlan K (2013). The effects of grazing on soil physical and chemical properties and plant diversity in North-Central Alberta. MSc Dissertation.

[ref-53] Mandal UK, Burman D, Mahanta KK, Sarangi SK, Shishir Raut, Subhasis M, Maji B, Bandyopadhyay BK (2015). Bulk soil electrical conductivity for coastal salt affected soils of West Bengal. Journal of the Indian Society of Coastal Agricultural Research.

[ref-54] Márquez-Madrid M, Gutiérrez-Bañuelos H, Bañuelos Valenzuela R, Muro-Reyes A, Valdez-Cepeda RD (2017). Macro-mineral concentrations in soil and forage in three grassland sites at Zacatecas. Revista Mexicana de Ciencias Pecuarias.

[ref-55] McCauley A, Jones C, Jacobsen J (2009). Soil pH and organic matter. Nutrient Management Module.

[ref-56] McLean EO (1983). Soil pH and lime requirement. Methods of Soil Analysis: Part 2 Chemical and Microbiological Properties.

[ref-57] Mengistu AG, Mavimbela SS, Van Rensburg LD (2019). Characterisation of the soil pore system in relation to its hydraulic functions in two South African aeolian soil groups. South African Journal of Plant and Soil.

[ref-58] Michopoulos P, Kostakis M, Thomaidis NS, Pasias I (2021). The influence of forest types on manganese content in soils. Folia Forestalia Polonica.

[ref-59] Miller R, Sonon L (2014). Nitrate-nitrogen. Soil test methods from the southeastern United States. https://www.clemson.edu/public/regulatory/ag-srvc-lab/soil-testing/index.html.

[ref-60] Minasny B, Hong SY, Hartemink AE, Kim YH, Kang SS (2016). Soil pH increase under paddy in South Korea between 2000 and 2012. Agriculture, Ecosystems & Environment.

[ref-61] Monreal CM, De Rosa M, Mallubhotla SC, Bindraban PS, Dimkpa C (2016). Nanotechnologies for increasing the crop use efficiency of fertilizer-micronutrients. Biology and Fertility of Soils.

[ref-62] Mucina L, Rutherford MC (2006). The vegetation of South Africa, Lesotho and Swaziland. Strelitzia 19.

[ref-63] Munyai FR (2012). An evaluation of socio-economic and biophysical aspects of small-scale livestock systems based on a case study from Limpopo Province. Doctoral dissertation.

[ref-64] Neina D (2019). The role of soil pH in plant nutrition and soil remediation. Applied and Environmental Soil Science.

[ref-65] Noellemeyer E, Quiroga AR, Estelrich D (2006). Soil quality in three range soils of the semi-arid Pampa of Argentina. Journal of Arid Environments.

[ref-66] Northup BK, Starks PJ, Turner KE (2019a). Soil macronutrient responses in diverse landscapes of southern tallgrass to two stocking methods. Agronomy.

[ref-67] Northup BK, Starks PJ, Turner KE (2019b). Stocking methods and soil macronutrient distributions in southern tallgrass paddocks: are there linkages?. Agronomy.

[ref-68] Oades JM (1993). The role of biology in the formation, stabilization and degradation of soil structure. Soil structure/soil biota interrelationships. Geoderma.

[ref-69] Olsen SR, Sommers LE, Page AL (1982). Phosphorus. Methods of soil analysis. Part 2: chemical and microbiological properties.

[ref-70] Parwada C, Van Tol J (2017). Soil properties influencing erodibility of soils in the Ntabelanga area, Eastern Cape Province, South Africa. Acta Agriculturae ScandInavica, Section B—Soil & Plant Science.

[ref-71] Penn CJ, Camberato JJ (2019). A critical review on soil chemical processes that control how soil pH affects phosphorus availability to plants. Agriculture.

[ref-72] Ragimov A, Mazirov M, Nikolaev V, Shitikova A, Malakhova S (2020). Impact of different type of cattle grazing on the processes of agrochemical degradation and digression of soil cover.

[ref-73] Rahmanian S, Hejda M, Ejtehadi H, Farzam M, Memariani F, Pyšek P (2019). Effects of livestock grazing on soil, plant functional diversity, and ecological traits vary between regions with different climates in northeastern Iran. Ecology and Evolution.

[ref-74] Raiesi F, Riahi M (2014). The influence of grazing exclosure on soil C stocks and dynamics, and ecological indicators in upland arid and semi-arid rangelands. Ecological Indicators.

[ref-75] Rapti D, Papaioannou AG, Parissi ZM, Karatassiou M (2016). Impact of different grazing intensities on rangelands soil characteristics in Central Greece. Options Méditerranéennes Series A.

[ref-76] Reeder JD, Schuman GE (2002). Influence of livestock grazing on C sequestration in semi-arid mixed-grass and short-grass rangelands. Environmental Pollution.

[ref-77] Reeder JD, Schuman GE, Morgan JA, LeCain DR (2004). Response of organic and inorganic carbon and nitrogen to long-term grazing of the shortgrass steppe. Environmental Management.

[ref-78] Rutherford MC, Powrie LW (2010). Severely degraded rangeland: implications for plant diversity from a case study in Succulent Karoo South Africa. Journal of Arid Environments.

[ref-79] Statistical Analysis System (2010). Statistics Software, Release 10.

[ref-80] Schiettecatte W, Gabriels D, Cornelis WM, Hofman G (2008). Enrichment of organic carbon in sediment transport by interrill and rill erosion processes. Soil Science Society of America Journal.

[ref-81] Schrama M, Heijning P, Bakker JP, Van Wijnen HJ, Berg MP, Olff H (2013). Herbivore trampling as an alternative pathway for explaining differences in nitrogen mineralization in moist grasslands. Oecologia.

[ref-82] Sebola MP, Mamabolo MA (2020). Governing and managing communal land as a resource in South Africa: a case of selected communal property associations in Vhembe district, Limpopo province. The Business and Management Review.

[ref-83] Selemani IS (2015). Influence of ngitili management on vegetation and soil characteristics in semi-arid Sukumaland, Tanzania. Livestock Research for Rural Development.

[ref-84] Semmartin M, Di Bella C, De Salamone IG (2010). Grazing-induced changes in plant species composition affect plant and soil properties of grassland mesocosms. Plant and Soil.

[ref-85] Sepehry A, Akhzari D, Pessarakli M, Barani H (2012). Studying the effects of salinity, aridity and grazing stress on the growth of various halophytic plant species (Agropyron elongatum, Kochia prostrata and Puccinellia distans). World Applied Sciences Journal.

[ref-86] Shi D, Wang D (2005). Effects of various salt-alkaline mixed stresses on *Aneurolepidium chinense* (Trin.) Kitag. Plant and Soil.

[ref-87] Shiferaw A, Yimer F, Tuffa S (2019). Changes in soil organic carbon stock under different land use types in semiarid Borana rangelands: implications for CO2 emission mitigation in the rangelands. Journal of Agricultural Science and Food Research.

[ref-88] Shrestha G, Stahl PD (2008). Carbon accumulation and storage in semi-arid sagebrush steppe: effects of long-term grazing exclusion. Agriculture, Ecosystems & Environment.

[ref-89] Shrivastav P, Prasad M, Singh TB, Yadav A, Goyal D, Ali A, Dantu PK (2020). Role of nutrients in plant growth and development. Contaminants in agriculture.

[ref-90] Šimek M, Cooper JE (2002). The influence of soil pH on denitrification: progress towards the understanding of this interaction over the last 50 years. European Journal of Soil Science.

[ref-91] Somenahally A, McLawrence J, DuPont JI, Brady J, Sarkar R, Rouquette Jr M (2020). Root-mycorrhizae interactions contributed to organic carbon density in the sandy soil profiles of adapted grazing lands. Applied Soil Ecology.

[ref-92] Steffens D, Sparks DL (1999). Effect of residence time on the kinetics of nonexchangeable ammonium release from illite and vermiculite. Journal of Plant Nutrition and Soil Science.

[ref-93] Steffens M, Kölbl A, Totsche KU, Kögel-Knabner I (2008). Grazing effects on soil chemical and physical properties in a semiarid steppe of Inner Mongolia (PR China). Geoderma.

[ref-94] Stichler C (2002). Grass growth and development. Texas Cooperative Extension.

[ref-95] Stuart Chapin III F, McFarland J, David McGuire A, Euskirchen ES, Ruess RW, Kielland K (2009). The changing global carbon cycle: linking plant–soil carbon dynamics to global consequences. Journal of Ecology.

[ref-96] Su YZ, Zhao HL, Zhang TH, Zhao XY (2004a). Soil properties following cultivation and non-grazing of a semi-arid sandy grassland in northern China. Soil and Tillage Research.

[ref-97] Su YZ, Zhao HL, Zhao WZ, Zhang TH (2004b). Fractal features of soil particle size distribution and the implication for indicating desertification. Geoderma.

[ref-98] Sun QB, Shen RF, Zhao XQ, Chen RF, Dong XY (2008). Phosphorus enhances Al resistance in Al-resistant Lespedeza bicolor but not in Al-sensitive L. cuneata under relatively high Al stress. Annals of Botany.

[ref-99] Tabatabai MA, Dick WA (1983). Simultaneous determination of nitrate, chloride, sulfate, and phosphate in natural waters by ion chromatography. American Society of Agronomy, Crop Science Society of America, and Soil Science Society of America.

[ref-100] Taddese G (2001). Land degradation: a challenge to Ethiopia. Environmental Management.

[ref-101] Tavakoli MT, Chenari AI, Rezaie M, Tavakoli A, Shahsavari M, Mousavi SR (2014). The importance of micronutrients in agricultural production. Advances in Environmental Biology.

[ref-102] Tripathi BM, Kim M, Singh D, Lee-Cruz L, Lai-Hoe A, Ainuddin AN, Go R, Rahim RA, Husni MHA, Chun J, Adams JM (2012). Tropical soil bacterial communities in Malaysia: pH dominates in the equatorial tropics too. Microbial Ecology.

[ref-103] US Salinity Laboratory US, Richards LA (1954). Diagnoses and improvement of saline and alkali soils. Agriculture handbook No. 60.

[ref-104] Van der Salm C, van den Toorn A, Chardon WJ, Koopmans GF (2012). Water and nutrient transport on a heavy clay soil in a fluvial plain in the Netherlands. Journal of Environmental Quality.

[ref-105] Van Oudtshoorn FP (2015). Veld management—principles and practices.

[ref-106] Van Oudtshoorn FP (2020). Guide to grasses of Southern Africa.

[ref-107] Van Rensburg LD, De Clercq WP, Barnard JD, Du Preez CC (2011). Salinity guidelines for irrigation: case studies from Water Research Commission projects along the lower Vaal, Riet, Berg and Breede Rivers. Water SA.

[ref-108] Van Wyk B, Van Wyk B, Van Wyk P (2012). Photo guide to trees of South Africa.

[ref-109] Vetter S (2013). Development and sustainable management of rangeland commons—aligning policy with the realities of South Africa’s rural landscape. African Journal of Range & Forage Science.

[ref-110] Walker B, Meyers JA (2004). Thresholds in ecological and social–ecological systems: a developing database. Ecology and Society.

[ref-111] Walkley A, Black IA (1934). An examination of the Degtjareff method for determining soil organic matter, and a proposed modification of the chromic acid titration method. Soil Science.

[ref-112] Waller NJ, Kookana RS (2009). Effect of triclosan on microbial activity in Australian soils. Environmental Toxicology and Chemistry: An International Journal.

[ref-113] Wang B, Waters C, Orgill S, Cowie A, Clark A, Liu DLi, Simpson M, McGowen I, Sides T (2018). Estimating soil organic carbon stocks using different modelling techniques in the semi-arid rangelands of eastern Australia. Ecological Indicators.

[ref-114] Wang Q, Batkhishig O (2014). Impact of overgrazing on semiarid ecosystem soil properties: a case study of the Eastern Hovsgol Lake Area, Mongolia. Journal of Ecosystem & Ecography.

[ref-115] Wang Y, Wesche K (2016a). Vegetation and soil responses to livestock grazing in Central Asian grasslands: a review of Chinese literature. Biodiversity and Conservation.

[ref-116] Wang Y, Wesche K (2016b). Vegetation and soil responses to livestock grazing in Central Asian grasslands: a review of Chinese literature. Biodiversity and Conservation.

[ref-117] Whitehead DC (2000). Nutrient Elements in Grassland: Soil-Plant-Animal Relationships.

[ref-118] Wilhelm SH (2009). Sulfur in soils. Journal of Plant Nutrition and Soil Science.

[ref-119] Yusuf HM, Treydte AC, Sauerborn J (2015). Managing semi-arid rangelands for carbon storage: grazing and woody encroachment effects on soil carbon and nitrogen. PLOS ONE.

[ref-120] Zhao XQ, Chen RF, Shen RF (2014). Coadaptation of plants to multiple stresses in acidic soils. Soil Science.

